# Effectiveness of tutorial videos combined with online classes in surgical knotting course during COVID-19 pandemic: A cohort study

**DOI:** 10.1016/j.amsu.2021.102751

**Published:** 2021-08-25

**Authors:** Adeodatus Yuda Handaya, Aditya Rifqi Fauzi, Joshua Andrew, Ahmad Shafa Hanif, Kevin Radinal Tjendra, Azriel Farrel Kresna Aditya

**Affiliations:** a Digestive Surgery Division, Department of Surgery, Faculty of Medicine, Universitas Gadjah Mada/Dr. Sardjito Hospital, Faculty of Medicine, Universitas Gadjah Mada/Dr Sardjito Hospital, Yogyakarta, 55281, Indonesia; b Faculty of Medicine, Universitas Gadjah Mada/Dr. Sardjito Hospital, Yogyakarta, 55281, Indonesia

**Keywords:** COVID-19 pandemic, Medical education online class, Surgical knotting, Tutorial video

## Abstract

**Background:**

COVID-19 pandemic has changed medical education from offline courses to online formats. Nowadays, offline skill demonstration lessons becomes unfeasible. This study assess the effectiveness of tutorial videos and online classes in delivering knowledge and skill in basic surgical knotting to medical students.

**Methods:**

A group of medical students (n = 95) was divided into two groups: the first group was allowed to watch the tutorial video that we have been made and uploaded into YouTube (https://www.youtube.com/watch?v=WyfOVGhAeVA) while the other group did not watch the video. All participants submitted a demonstration video to show their knotting skill. These videos were graded and made into the first evaluation. Then, all participants attended online classes for the surgical knotting skills via Zoom application. Participants submitted another demonstration video after the online classes. The videos were assessed, and the results were analyzed.

**Results:**

The experimental group (n = 50) who watched the tutorial video prior to class scored higher in the first video than the control group (n = 39) with a meanscore of 10.850 versus 7.462, *p* = 0.000*, In the second video, the assessment showed no significant difference between the two groups with meanscore of 11.220 versus 10.897, *p* = 0.706.

**Conclusion:**

The combination of tutorial videos and online classes is the optimal teaching method for surgical knotting skills.

## Highlights

• E-learning becomes a favorable learning method in COVID-19 pandemic era.
• Combination of tutorial video and live online skill demonstration is proven effective in teaching surgical knotting skills to medical students.
• Recording individual skill and individual feedback sessions is the optimal teaching method for surgical knotting skills.

## 1 Introduction

Since early 2020, COVID-19 pandemic has impacted many life sectors, including education. Many universities and schools have shifted from on-site classes to online formats to accommodate physical distancing safety protocols. One of the challenges faced by medical education institutions was how to train the procedural skill subjects without the traditional live demonstrations and directly observed skills laboratory. Also in early 2020, Indonesian government imposed a wide scale social restrictions to limit the acceleration of COVID-19 transmission, therefore forbidding on-site learning in school and universities [1]. This makes e-learning as the only feasible choice to continue education in this pandemic setting [2].

Some studies showed that video education is at least as good as the traditional methods in teaching medical students. For example, Pilieci et al. found that video education is superior to traditional skill demonstration in providing medical students with knowledge of sterile surgical techniques. McKenny et al. found that video education approaches were as effective as direct demonstrations in teaching nursing students how to change nursery dressing. Additionally, Nageswaran et al. found that the addition of video is better than traditional skills sessions alone, while Weber et al. found that the use of video-based instruction to teach surgical hand disinfection is more effective, efficient, and acceptable than conventional instruction [3,4].

A good surgical knot is important for loop security to ensure good tissue attachment and healing. A loose surgical knot may result in many complications, such as hemorrhage, anastomotic leakage, incisional hernia, impaired in tissue healing, and poor cosmetic appearance [5,6]. However, a tight surgical knot may result in tissue ischemia leading to impaired healing and leakage. Furthermore, having too many knots may increase tissue reaction, and cause prolonged inflammation, thus impairing wound healing, and making a bulging scar.

Many studies presented the advantages and disadvantages of e-learning in general such as using video conference through zoom application. The comparison between the effectiveness of online learning and offline learning has also been reported in few studies. However, our study is slightly different as we observe the effectiveness of online learning in teaching medical students basic surgical knotting. We want to determine wether the combination of online class and tutorial video method in the COVID-19 pandemic setting can be feasible and effective in teaching surgical skills that conventionally taught directly in class or skills laboratory session. This study in line with STROCSS 2019 guideline [7].

## 2 Material and methods

A video of basic surgical knotting tutorial was created and uploaded to YouTube (can be accessed at: https://www.youtube.com/watch?v=WyfOVGhAeVA) by the author as a digestive surgeon, instructor in charge, and the Basic Suturing Skill Course Director in Indonesia. All the steps and methods are based on the Royal College of Edinburgh standards.

This study was a cohort prospective single centered study. We observed 95 medical students in the Surgery Department clinical rotation of Universitas Gadjah Mada/Dr. Sardjito Central Hospital in this study. Basic surgical knotting is part of the education curriculum. The students have learned the theory of knotting in undergraduate program but lack experience in the clinical setting. They were divided into 2 groups: the experimental group (n = 53) who were given the knotting video prior to the first task, and the control group (n = 42) who were not. All students made a first knotting video before attending online classes with the instructor. After the class, participants made another knotting video. All videos were submitted through Google forms. For the videos, all participants may use any type of ropes and objects that can become core of the knot.

This study was approved by the Institutional Review Board of the Faculty of Medicine, Public Health and Nursing, Universitas Gadjah Mada/Dr. Sardjito Hospital, Yogyakarta, Indonesia (KE/FK/0849/EC/2021).

The inclusion criteria of this study were: 1) participant was enrolled in surgery clinical rotation, and 2) participant submitted both pre-class and post-class videos. Whereas the exclusion criteria was: 1) the video file was not complete or corrupted in some way. This study was double-blinded. Video assessment was done by two assessors, and the instructor in charge as certified Basic Suturing Skill Course Director in Indonesia.

The analyses were made with SPSS Statistics 23 (IBM Corp., Armonk, NY). Data were tested for normality with Kolmogorov Smirnov test. Since the data were not distributed normally, the Mann-Whitney test was used to compare both groups and Wilcoxon test to compare pre and post results between each group.

## 3 Results

**Fig. 1:**
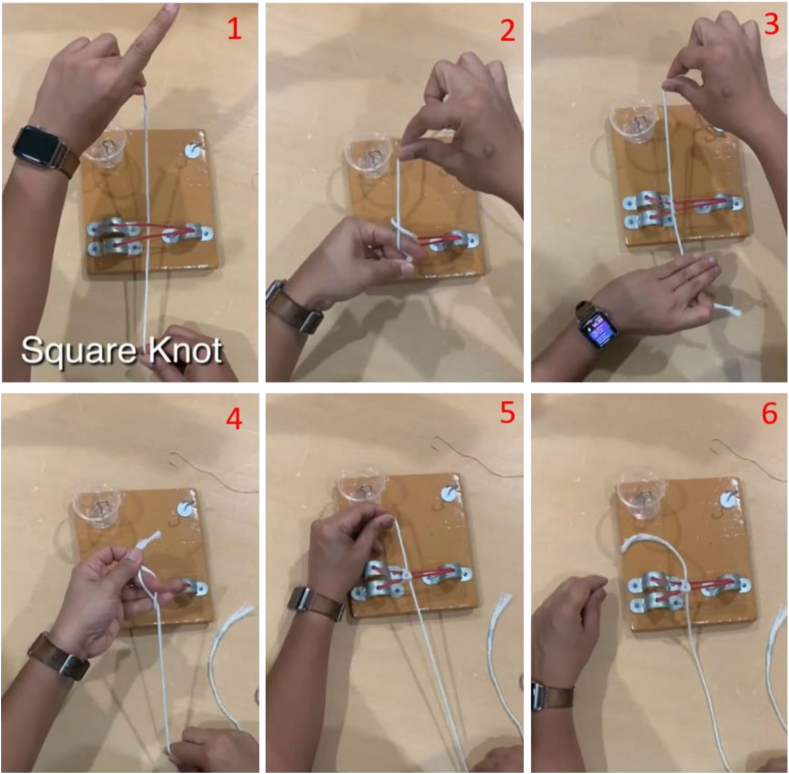
Surgical knotting steps demonstrated in video.

The video consisted of a step-by-step demonstration to make surgical knotting. The steps shown in video are included in Fig. 1 below and each step were scored as: 0 if the step was not done; 1 if partially done; and 2 if correctly done. The total score was compared in this study (Table 1) (Fig. 1)

**Table 1:** Steps and score checklist.

Steps	
1	Left hand acts the primary movement
2	Loop formation
3	First throw execution
4	Square knot or half surgeon knot formation
5	Second throw execution
6	Quality of the knot (firm not loose)

**Fig. 2:**
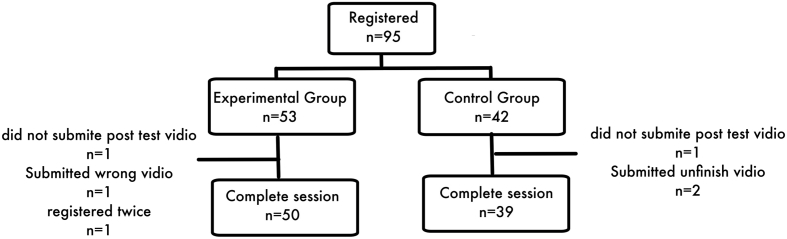
Participant distribution.

The participants were 95 students registered to the surgery clinical rotation in July to October 2021. The July (n = 26) and August (n = 27) groups were the experimental group whereas the September (n = 24) and October (n = 18) groups were the control group. From the experimental group, 2 participants were not included because 1 participant did not submit the post-class video and 1 participant registered twice, while1 participant was excluded since the post-class video submitted was not surgical knotting. From the control group, 1 participant was not included since the post-class video was not submitted and 2 participants were excluded because the post-class videos were cut and appeared edited (Fig. 2).

In the comparison of the control and experimental groups fore pre-class videos, we found significant differences in the total scores with *p*-value of 0.000. However, there was no significant difference in post-test comparison with *p*-value of 0.706 (Table 2).

**Table 2:** Control and experimental group comparison.

	Control Group			Experimental Group			p – Value
	n	Mean	Std Deviation	n	Mean	Std Deviation	
Pre-Test	39	7.462	1.7259	50	10.850	1.8077	0.000*
Post-Test	39	10.897	1.9808	50	11.220	1.5358	0.706

When comparing the pre-class and post-class results in the control and experimental groups, we found differences in each group with *p*-value of 0.000 and 0.001, respectively (Table 3).

**Table 3:** Pre-test and post-test comparison.

	Pre-Test			Post Test			p – Value
	n	Mean	Std Deviation	n	Mean	Std Deviation	
Control Group	39	7.462	1.7259	39	10.897	1.9808	0.000*
Experimental Group	50	10.850	1.8077	50	11.220	1.5358	0.001*

**Fig. 3:**
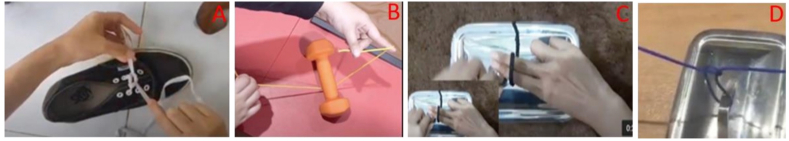
Screenshot sample of participants' video. (A) A good square knot done in shoe media. (B) Loop formation in fitness dumbbell media. (C) Wrong technique application, the participant demonstrating instrument knotting in instrument box media. (D) Wrong loop formation, the loop was crossed, thus increasing the risk of snapped thread.

The following figures showed knotting media used by participants in this study. The majority of participants used household appliances (n = 51, 57%), such as shoes, chair, rubber band, table, etc., followed by medical instruments (n = 38, 43%), especially stainless-steel instrument set box (Fig. 3).

## 4 Discussion

Since the World Health Organization (WHO) declared the COVID-19 outbreak as a global pandemic in late 2019, the restrictions in people's movement to control the spread of the virus have affected all life aspects, especially education, including medical education. As social distancing was adopted, on-site classes and lectures were suspended. Meanwhile, the delayed graduations, especially in medical education, may have negative impacts, especially in this pandemic management setting. Therefore, educational methods have been shifting to online approaches [2,8,9].

Online teaching itself has been important in medical education in recent years and has been shown to be on par with offline teaching in examination results [2]. The advantage of online learning is the learning process is learner oriented. Students are able to study anytime, anywhere, following their own pace, and repeating specific parts they wanted to review, all according to their own ability and preferences [3,10,11]. Online learning also enables various multimedia content to be used. Online learning processes have many interactive and virtual platforms, such as live webinar, teleconference, educational applications, or educational videos which all can be accessed through various gadgets including laptops and Smartphones [3,12].

However, there are some challenges in online medical education, namely student's lack of time management, uneven ability to access and gadgets between students, communication and interaction between lecturers and students, and difficulty in students' assessments [11,13]. The effectiveness of online education is also influenced by the learners' individual characteristics since differences in gender, learning style, attitude, satisfaction, and engagement can contribute to differences in the outcomes [14]. Some learning contexts are also difficult to shift to online learning, especially for psycho-motor skills, including surgical knotting. This kind of topic requires teacher-student interaction and feedback which can be challenging [9].

Basic surgical skills, including surgical knotting, incision, and suturing are normally learned in on-site class and skills laboratory settings, mentored face-to-face by a skilled surgeon [9,12,15]. Research by Wang et al. showed that conventional self-regulated learning and guided video reflection are equal in teaching basic psycho-motor skills and the use of guided video reflection improved self-assessment [15]. In previous study, the use of video learning also helped medical students in clinical examination, suturing ability, and overall intraoperative learning [3].

Participants are allowed to use any kind of rope and other household material to demonstrate the surgical knotting skill. A proper mannequin and knotting media could not be used since the availability of those media is limited to the skills laboratory on the campus. However, the diversity of media, as shown in the above figures, in this study did not hinder the results because the point of the session was the knot formation.

In our study, we found that students who watched video lessons of basic surgical knotting excelled in the pre-class examination more than students who did not. After participating in the online class there was no difference in scores for the post-class video assessment between the two groups. This shows that online class is an effective method in teaching surgical knotting skills regardless of prior knowledge and skills. The classes, which are comprised of lectures, live demonstration of skills, and individual feedback sessions, cannot be replaced by tutorial video alone.

When looking at the result alone, we can infer that live online classes can give the optimal learning for knowledge and surgical skills. However, in real practice, we believed students that have prior exposure to the tutorial video have less difficulties in following the class and in the end, will produce or replicate the surgical skill with more ease than the students that have no prior knowledge. Our inference in this issue varies and may differ from several studies such as a study by Pilieci et al. which stated that tutorial videos are better than live demonstration of surgical skills [2]. Tutorial videos may have many advantages such as repeatable and can be paused or played slower for better observation of the procedures. We strongly believed that online class can be more beneficial since it allows interaction and discussion between the teacher and the students for better understanding. Despite the many benefits of tutorial videos, it should not be the only learning material that is provided to the students. Therefore, tutorial videos should be followed with online classes to provide better understanding.

## 5 Conclusions

The combination of tutorial videos and online classes comprising of lectures, live demonstration of skills, and individual feedback sessions is the optimal teaching method for surgical knotting skills.

## Ethical approval

This study was approved by Universitas Gadjah Mada Ethical Committee with Ethical Clearance KE/0686/06/2021. This study was registered in Universitas Gadjah Mada research repository with register ID 202107100.

## Funding

This research did not receive any specific grant from funding agencies on the public, commercial, or non-for-profit sectors.

## Author’s contribution

Adeodatus Yuda Handaya conceived the study. Joshua Andrew drafted the manuscript. Aditya Rifqi Fauzi, Ahmad Shafa Hanif, Kevin Radinal, and Azriel Farrel Krisna Aditya critically revised the manuscript for important intellectual content. All authors read and approved the final draft.

## Registration of research studies

Research Repository Faculty of Medicine, Public Health and Nursing, Universitas Gadjah Mada Register ID: 202105099.

## Guarantor

Adeodatus Yuda Handaya is the guarantor of the study.

## Declaration of competing interest

None.
